# Regular, narrow QRS, long RP tachycardia – what is the mechanism?

**DOI:** 10.1007/s12471-016-0898-3

**Published:** 2016-09-16

**Authors:** S. Tzeis, S. Pastromas, A. Sikiotis, G. Andrikopoulos

**Affiliations:** Pacing and Electrophysiology Department, Henry Dunant Hospital Center, Athens, Greece

We report the case of a 45-year-old male patient who was referred to our department due to palpitations for the last six months, with documented episodes of regular, long RP, narrow QRS tachycardias (Fig. 1). The patient had no history of structural heart disease, his transthoracic echocardiography demonstrated normal systolic and diastolic function and the thyroid function tests were within normal limits. The 24-hour Holter monitoring showed multiple, recurrent, self-terminating episodes of regular, long RP tachycardias (Fig. 2).

**Fig. 1:**
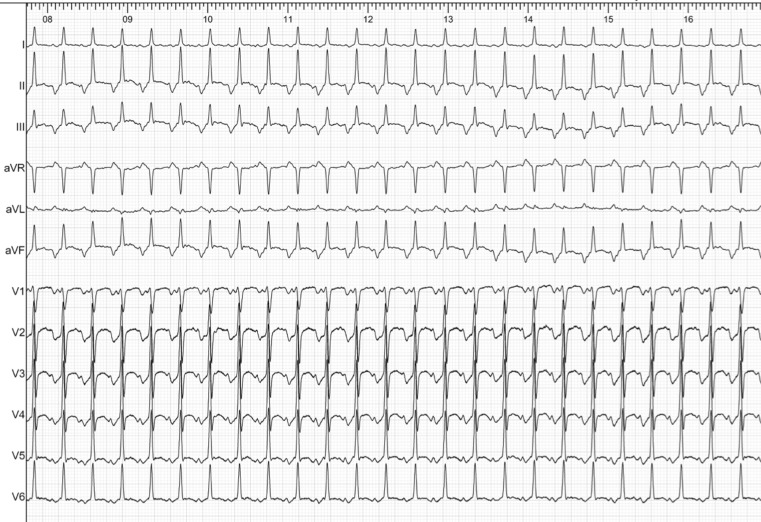
Twelve-lead surface ECG of the index regular, long RP, narrow QRS tachycardia

**Fig. 2:**
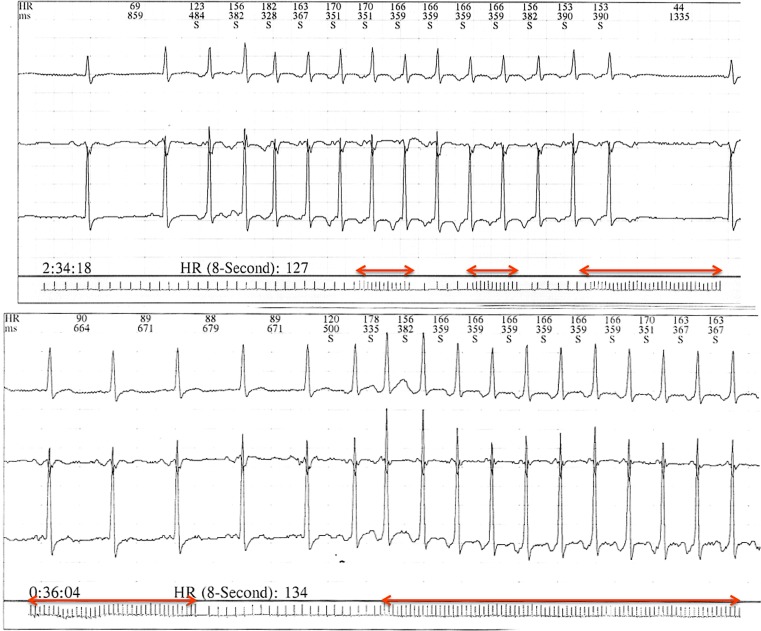
Tracings from the Holter recording showing repetitive, self-terminating episodes of long-RP tachycardia. The horizontal arrows show the duration of the recurrent bouts of the tachycardia during rhythm recording

The patient was referred for an electrophysiological study. During the procedure, the patient was almost continuously in a regular long-RP tachycardia. Any attempt of either atrial or ventricular pacing during intermittent sinus rhythm resulted in reproducible tachycardia induction, which precluded the evaluation of the retrograde conduction properties. Therefore, only pacing manoeuvres during the index tachycardia could be used to differentiate the underlying mechanism.

What is the differential diagnosis of the index tachycardia?

Which diagnostic manoeuvre during the electrophysiological study is suggested for the delineation of the underlying tachycardia mechanism?

## Answer

You will find the answer elsewhere in this issue.

